# Is annual surveillance of all treated hypothyroid patients necessary?

**DOI:** 10.1186/1472-6823-7-4

**Published:** 2007-07-02

**Authors:** Ananth K Viswanath, Alison Avenell, Sam Philip, Shamsunder H Acharya, Graeme MacLennan, Katherine Dalziel, Olivia Pereira, Shirley A Copland, John S Bevan, Prakash Abraham

**Affiliations:** 1 Thyroid clinic, Aberdeen Royal Infirmary, Aberdeen AB25 2ZN, UK; 2 Health Services Research Unit, Health Sciences Building, University of Aberdeen, Foresterhill, Aberdeen AB25 2ZD, UK; 3 Department of E-Health, NHS Grampian, Aberdeen AB25 2HX, UK

## Abstract

**Background:**

Annual surveillance (with thyroid function testing) is widely recommended for the long-term follow-up of treated hypothyroid patients. It is based largely on consensus opinion and there is limited evidence to support the frequency of monitoring. The majority of patients in our hospital based thyroid register are on 18 monthly follow-up.

**Methods:**

We carried out a retrospective analysis to see if there is evidence to support more frequent testing. We used a logistic regression model to assess whether any baseline characteristics could be applied to predict an abnormal test.

**Results:**

We identified 2,125 patients with a minimum of 10 years follow-up (89% female, 65% autoimmune hypothyroidism, and mean age at registration 51 years). There were 2 groups: 1182 (56%) had been allocated to 18 monthly follow-up and the rest had annual surveillance. The groups were well matched at baseline. Overall, during follow-up the 12 monthly group had more abnormal tests requiring dose adjustment. However, on logistic regression analysis, people aged less than 60 years, individuals taking < 150 μg thyroxine per day and people on 18 monthly follow-up had less abnormal tests.

**Conclusion:**

18 monthly surveillance may be adequate in the long term follow-up of hypothyroid patients less than 60 years of age on a stable thyroxine dose of 100–150 μg/day where there are robust follow-up mechanisms in place. Implementing this strategy has potential for cost saving.

## Background

Hypothyroidism is usually due to primary thyroid failure secondary to chronic autoimmune thyroiditis (Hashimoto's disease) or destructive therapy (radioactive iodine or thyroidectomy). In the United Kingdom the prevalence of overt hypothyroidism in women is at least 1.4 to 1.9% compared to less than 1% in men [1], and increases with age [2]. Levothyroxine is the standard replacement therapy and treatment is life long, with the aim of restoring patients to a euthyroid state and normalising thyroid stimulating hormone (TSH) concentration. Though the treatment of hypothyroidism is straightforward, data from several studies [3-5] show that only around 60% of patients on thyroxine replacement have normal TSH levels. Thyroxine over replacement is associated with increased risk of atrial fibrillation [6] and reduction in bone mass in postmenopausal women [7]. In addition a raised TSH level is associated with an unfavourable lipid profile [4,8].

Hence periodic monitoring of thyroid function tests (TFTs) is essential in the management of patients to judge the response to therapy, patient compliance, and to adjust doses in relation to advancing age. Annual TFT surveillance is widely recommended [9-11]. In the UK, the General Practitioner (GP) contract [12] effective from April 2004, which financially rewards primary care practices, recommends that patients with hypothyroidism on thyroxine, should have thyroid function tests recorded at least every 15 months. The recommended frequency of testing is based largely on consensus, and there is limited evidence to support this. The majority of patients currently followed up in our hospital based thyroid register are on 18 monthly follow-up. We undertook a retrospective study to see if there is evidence to support more frequent testing.

## Methods

Grampian Automated Thyroid Register (previously known as Scottish Automated Follow-up Register) has provided follow-up facilities for patients with thyroid disease since 1967, as a joint venture between hospital and primary care. Currently around 20,000 patients are registered on the database. Patients are recalled at a pre-set follow-up interval for TFTs and review by the primary care staff, who provide information on clinical status. The results are reviewed centrally, and if satisfactory, the computer automatically generates a routine recall. If abnormal, the result is reviewed by a thyroid specialist who may recommend a dose change and/or an early review. Written instructions are sent to the GP, reducing the number of patients with abnormal tests requiring review at a hospital based thyroid clinic. After an appropriate intervention, when TFTs are satisfactory, individuals are returned to routine review at the pre-set interval. If they fail to turn up for review, a reminder is sent at 3 and 6 months. Deaths are notified to the thyroid register.

We retrospectively identified hypothyroid patients on thyroxine who had a minimum of 10 years of follow-up on the register. Only patients on 12 monthly and 18 monthly follow-up plans were evaluated. As there were no set guidelines for entering patients on the register, patients were allocated to these follow-up intervals by clinicians on a non-selective basis. We excluded patients who hadn't returned for follow-up for more than 30 months (patients who may have moved away from the region or were non-compliant). Patients with drug-induced hypothyroidism and 11 individuals with inadequate follow-up data were also excluded from the study. The registration details included age, aetiology of hypothyroidism and thyroxine dose at registration. Follow-up information regarding the number of reviews, defaults, changes in thyroxine dose or follow-up interval and the results of the thyroid test at each review was summarised from the database. TFTs undertaken during follow-up were interpreted as hyperthyroid when TSH was suppressed (<0.01 mU/L) and other thyroid hormones were elevated. An elevated TSH (>4 mU/L) with or without a low thyroid hormone concentration was described as a hypothyroid test. The method used for thyroid function testing varied during the study period. Radio-immuno assay (RIA) was used up to1995 (reference range: total T4 70–150 nmol/L, TSH 0.35–3.3 mU/L). From 1995 onwards automated immunoassay was used (Bayer diagnostics, reference range: FT4 10–25 pmol/L, TSH 0.35–3.3 mU/L). Data were anonymised and permission was not required from the Grampian Regional Ethics Committee to carry out this research.

### Statistical methods

Data were analysed using Student's t-tests, Mann Whitney and chi squared tests as appropriate. To explore the differences in abnormal tests between the follow-up plan logistic regression was used. Covariates adjusted for were gender, autoimmune hypothyroidism (yes or no), age at registration (<25 years, 25 to 60 years, >60 years), thyroxine dose at registration (<100 μg, 100 to 150 μg, >150 μg), thyroid follow-up plan (12 monthly or 18 monthly) and duration of follow-up in years. All analyses were carried out in SPSS.

## Results

### Patient demographics

2,125 patients satisfied the inclusion criteria and the majority (89%) were female. The mean age at registration was 50.9 (standard deviation, SD, 13.3 years). The majority (65%) had developed spontaneous (autoimmune) hypothyroidism. Other causes included post radioiodine treatment (23.0%), post surgery (6.6%), thyroiditis (0.7%), and unavailable in the rest (4.7%). 1182 (55.6%) were on 18 monthly follow-up and the rest had annual surveillance. There was no significant difference between the groups in relation to the baseline characteristics age, sex and thyroxine dose (Table 1). A slightly higher proportion of patients on 18 monthly follow-up (68% versus 62%) had autoimmune hypothyroidism (p < 0.01).

**Table 1:** Comparison of baseline characteristics between the groups

**Baseline characteristics**	**12 monthly FU (n = 943)**	**18 monthly FU (n = 1182)**
**Age years **mean (SD)	50.2 (13.9)	51.6 (12.8)
**Thyroxine dose at registration (μg/day) **mean (SD)	131.8 (41.4)	130.7 (43.1)
**Age < 60 years **(n = 1556)	73.4%	73.1%
**Female **(n = 1892)	90.3%	88.0%
**Autoimmune hypothyroidism **(n = 1384)	61.6%	67.9%
**Mean duration of follow-up (years) **mean (SD)	16.5 (4.6)	15.6 (4.4)

### Follow-up

The average duration of follow-up was 16.2 (SD 4.5) years (range 10–30). Comparisons between the two groups are summarised in Table 2. As expected the 12 monthly follow-up group required more reviews. Both groups were largely compliant with follow-up, with more than 90% attending a regular review as scheduled. Only a small minority defaulted and had late reviews. A slightly higher proportion of individuals in the 18 monthly group required a review earlier than scheduled (usually at 6 months) compared to the 12 monthly group (6.2% vs 5.0%). The 12 monthly group had significantly more thyroid function tests compared to the 18 monthly group (16,700 vs 14,526, p < 0.01) during follow-up.

**Table 2:** Comparison of follow-up data between the groups

**Follow-up data**	**12 monthly (n = 943)**	**18 monthly (n = 1182)**
**Total number of reviews**	**15,191**	**13,864**
**Percentage of early reviews**	5%	6.2%
**Percentage of late reviews**	4.4%	2.4%
**Percentage of routine reviews**	90.6%	91.4%
**Total number of tests**	**16,700**	**14,526**
**Average number of tests per person**	17.7	12.3
**Percentage of patients with at least one abnormal test during follow-up**	**81%**	**73%**

### Thyroxine dose changes

The mean thyroxine dose at the time of registration was 131.2 (SD 42.4) μg per day (range 25–300) and around 80% were on 100–150 μg of thyroxine per day. The mean thyroxine dose at last/final review was 127.4 (SD 35.5) μg (median 125, range 25–300). During follow-up, thyroxine dose remained unchanged in 35.4%, and a further 31% and 21% of cases required a dose alteration of 25 μg and 50 μg respectively during long term surveillance. Individuals taking 100–150 μg of thyroxine at registration had fewer dose changes compared to the rest (40.5% vs 14.7%, p < 0.01). Only 62.7% on 18 monthly follow-up required a dose change as opposed to 66.9% on annual surveillance (p < 0.05).

### Outcomes

Abnormal test results were defined as both hyperthyroid and hypothyroid results. Seventy three percent on 18 monthly follow-up had at least one abnormal test during long term follow-up as compared to 81% on annual surveillance (p < 0.01). We used a logistic regression model to assess if any baseline characteristics could be applied to predict an abnormal test. The results are summarised in Table 3. Older patients (aged >60 years) at registration were more likely to have an abnormal test during follow-up (odds ratio 1.36, 95% CI 1.07–1.73, P < 0.01). In relation to thyroid screening interval, the 18 monthly group was less likely to have an abnormal test compared to the 12 monthly group (odds ratio 0.66, 95% CI 0.53–0.81, P < 0.01). There was no interaction between the sub-group variables and the screening interval. In addition, there was no difference in mortality between the 2 groups.

**Table 3:** Predictors of abnormal result

**Baseline Characteristics**	**Odds ratio**	**95% confidence interval**	**P value**
Male	0.81	(0.59, 1.13)	0.21
Autoimmune hypothyroidism	0.98	(0.79, 1.22)	0.86
*Age at registration:			
< 25 yrs	0.96	(0.54, 1.70)	0.96
> 60 yrs	1.36	(1.07, 1.73)	0.01
**+Thyroxine dose at registration**			
<100 μg	1.34	(0.86, 2.09)	0.18
>150 μg	1.36	(0.98, 1.90)	0.07
**Thyroid FU plan**			
18 monthly interval	0.66	(0.53, 0.81)	<0.001

*Reference category: 25 to 60 years; +Reference category: 100 to 150 μg

## Discussion

It is universally accepted that patients on thyroxine replacement should have regular clinical and biochemical surveillance to ensure compliance and adjust doses in relation to changing requirements. However the ideal screening interval has not been ascertained and there are no published studies on the appropriate frequency of follow-up. Surveillance by annual TFT is recommended by most of the published thyroid guidelines [9-11]. The recommendations are largely based on consensus, and there is limited evidence to support this practice. Vanderpump et al [9] recommended further audit/study to clarify some of the uncertainties in the follow-up of hypothyroid patients.

Grampian Automated Thyroid Register (GAFUR) was established in 1967 [13], and is one of the oldest thyroid registers in the world. Thyroid registers improve records, ensure regular reviews, and promote early detection of changes in the patient's clinical status. Thyroid registers have proved to be beneficial in the long-term follow-up of patients with thyroid dysfunction. They are cost-effective [14] and achieve better biochemical control [15] in comparison to conventional follow-up. From our thyroid register we were able to identify a large cohort of hypothyroid patients on thyroxine with a minimum of 10 years of follow-up. As expected the majority of patients were women who had developed spontaneous hypothyroidism. As there were no set guidelines, individuals were allocated to either follow-up plan by the referring physician on a non-selective basis. The baseline characteristics were well matched and there did not appear to be any bias in relation to allocation to either group. The system of recall and follow-up was well managed through good co-operation and links between primary care and GAFUR. Individuals in both the groups were compliant with follow-up arrangements. Similar benefits of thyroid registers in maintaining long term surveillance with minimal defaults was reported by Jung et al. in 1991[16].

During follow-up thyroid function tests were undertaken at each review appointment and 18 monthly follow-up was not associated with an increase in adverse outcomes. On the other hand, patients on annual surveillance were more likely to have an abnormal test. Both the groups were well matched at baseline (table.1) in relation to age and sex distribution, thyroxine dose and duration of follow-up, and we cannot readily account for the difference in outcome. One possible explanation could be physician bias at the time of registration. There were no set guidelines or criteria and it is possible that there was physician bias while allocating individuals to annual screening taking into account factors such as compliance. It is also likely that more frequent testing will find more abnormal results. Another conclusion that can be drawn is that annual surveillance should be recommended as there was an increased chance of detecting an abnormal result. This approach may however lead to unnecessary tests in some individuals as thyroxine dose remained unchanged in 35% of patients during long term follow-up. On the other hand, although 18 monthly surveillance cannot be applied universally, it may be an option in individuals whose thyroxine requirement is less likely to change or have less abnormal tests during follow-up. We undertook further sub-group analysis to identify individuals who may be suitable for less frequent testing.

Patients aged over 60 years at the time of registration were more likely to have an abnormal test during follow-up. This may be due to decreased thyroxine requirements with increasing age. This has been reported in previous studies [17,18] and probably reflects the progressive decrease in thyroxine degradation rate that occurs with advancing age. The fall in thyroxine requirement with age also corresponds to the loss of lean body mass [19]. Hence people aged over 60 years require annual surveillance.

Individuals taking 100–150 μg/day required fewer dose changes during long term follow-up compared to the rest. We would recommend annual testing in individuals who are on either <100 or >150 μg of thyroxine per day. Once established on the appropriate dose of thyroxine, follow-up interval can be extended to 18 months in individuals aged less than 60 years. There were insufficient patients on the register with a regular follow-up interval of two years to evaluate even less frequent monitoring.

Individuals on annual surveillance had more frequent reviews and required more TFTs compared to the 18 monthly group, and this will have cost implications. The Grampian automated thyroid register currently has over 10,000 registered hypothyroid patients, and the majority are being followed-up 18 monthly. Allocating everyone to annual follow up would mean an extra 3,000 thyroid tests per year. A thyroid function test costs around £15 for the NHS (personal communication with Dr Heather Watson, clinical biochemistry, NHS Grampian on 24/04/06). This would amount to an extra spending of around £45,000 per year without taking into account the cost of additional administrative time for GAFUR and primary care, and inconvenience to the patient. Our study has shown that 18 monthly follow-up may be adequate in the majority of patients under 60 years of age on a stable thyroxine dose of 100–150 μg/day. Implementing these recommendations has potential benefits in terms of savings and reduction in the workload.

The Grampian automated thyroid register was set up in the late 1960s and traditionally TSH and FT4 estimations were undertaken. The recently published UK guidelines [20] on the use of thyroid function tests recommend TSH estimation alone in the long-term follow-up of treated hypothyroid patients. We are considering changing our practice to achieve additional cost savings in relation to thyroid tests.

## Conclusion

Treated hypothyroid patients require regular clinical and biochemical surveillance. Annual surveillance is currently recommended with no evidence to support this practice. Our study has shown that 18 monthly surveillance may be adequate in the long term follow-up of patients less than 60 years of age on a stable thyroxine dose of 100–150 μg/day. Implementing this strategy has potential for cost saving and reduction of workload in health services.

## Competing interests

The author(s) declare that they have no competing interests.

## Authors' contributions

AKV contributed to the design of the study, the analysis and interpretation of data, and wrote the manuscript. AA contributed to the design of the study, guided data collection, supervised data analysis and critical review of manuscript. SP contributed to conception and design of the study, designed and implemented the algorithms for data extraction from the register. SHA helped in interpreting results and drafting manuscript. GM helped with statistical analysis and interpretation of data. KD handled the database, ran queries and helped with data collection. OP conceived and designed the initial pilot study. SAC and JSB provided advice through the study and critical review of manuscript. PA conceived and designed the study, guided data collection, supervised data analysis, critical review of manuscript. All authors have read and approved the final manuscript.

## Pre-publication history

The pre-publication history for this paper can be accessed here:


